# Commentary: Implementing Pro-Poor Universal Health Coverage

**DOI:** 10.3389/fpubh.2016.00186

**Published:** 2016-08-29

**Authors:** Mihajlo Jakovljevic

**Affiliations:** ^1^The Faculty of Medical Sciences, University of Kragujevac, Kragujevac, Serbia; ^2^Hosei University Tokyo, Tokyo, Japan

**Keywords:** universal health coverage, low middle income countries, risk sharing, financial, BRICS, global health

Recently published extraordinary article entitled: “implementing pro-poor universal health coverage” depicts an issue of truly global outreach (1). Modern day health system establishments had their historical roots back in the early industrial era of late nineteenth century Europe (2). Risk sharing through introducing the first health insurance funds was initially targeted to protect industrial laborers as an important segment of the society of the time (3). Health coverage of citizens beneath poverty line therefore began to slowly expand to the other vulnerable groups. During the first half of twentieth century, such practice spreads to North America (4) and Japan (5). It is less known that the first nationwide success in achieving universal health coverage (UHC) is attributable to the early Soviet Union back in 1930s and its famous Semashko system (6). Disintegration of colonial system worldwide after the end of WWII and rise of the non-aligned movement gave significant impetus to the health system developments among the Third World nations (7). After the end of Cold War Era, accelerated pace of globalization saw the uneven growth of welfare in these countries (8). Although attractive as a policy goal, health coverage for massive rural populations remained a distant dream for many world regions (9).

The aforementioned paper by Bump et al. pointed out to the core global UHC developments in a comprehensive manner. Their call to national governments to commit to the established milestones of UHC evolution is clear and might indeed serve the purpose. Nevertheless, few crucial facts were omitted, which might significantly narrow the horizon of perception on global evolution of UHC with regard to the role of BRICS nations (10).

Due to overall increase in welfare, UHC for the poor rapidly expanded around the world (11). Therefore, it seems that we might be deceived by perception that all of these world regions contributed evenly or at least to the comparable extent (12). The reality is rather different: there is a very narrow circle of top emerging economies to which we own most of this progress. Lion share of the growth in UHC, the world owns to the BRICS nations (13). Accounting for roughly two-fifths of world’s population, over the past two decades these national governments lifted from poverty hundreds of millions of the world’s poorest citizens (14). Quite efficient government policies dedicated to reducing poverty took place in these economies since late 1990s with few notable examples led by Chinese overachievement (15–17). Such an increase in social welfare of poorest citizens was attributable to industrial enterprise and direct foreign investment (18, 19). Their health reforms were bold and successful to the large extent leading to the notable gains toward achieving UHC (20). Distinctive role of these economies in global health arena led WHO Bulletin to establish a specialty issue committed to BRICS back in 2014 (21). Some of the exposed weaknesses alongside this ambitious process were India’s inability to expand health expenditure in terms of GDP percentage (22). Socioeconomic inequalities in health care expanded in some members of the group driven by exploding prevalence of prosperity diseases (23). Despite the fact of these obstacles accelerated expansion of UHC remains clearly visible in Russia, Brazil, India, and China (24). One of the surprising developments is the strong and continuing upward trend of their national abilities to increase investment in health care and expand insurance coverage of the population below poverty line (25). Long-term commitment of BRICS governments ultimately resulted in significantly improved health outcomes, including nationwide longevity (26). A global landscape of UHC evolution implies that orchestrated international efforts should regard these nations as one of the pillars of any responsible policy aimed to protect the world’s poor from health-related risks.

## Author Contributions

MJ has designed drafted and finalized the manuscript.

## Conflict of Interest Statement

The author declares that the research was conducted in the absence of any commercial or financial relationships that could be construed as a potential conflict of interest.
